# Stress-Induced and Diabetic Hyperglycemia Associated with Higher Mortality among Intensive Care Unit Trauma Patients: Cross-Sectional Analysis of the Propensity Score-Matched Population

**DOI:** 10.3390/ijerph15050992

**Published:** 2018-05-15

**Authors:** Meng-Wei Chang, Chun-Ying Huang, Hang-Tsung Liu, Yi-Chun Chen, Ching-Hua Hsieh

**Affiliations:** 1 Department of Emergency Medicine, Kaohsiung Chang Gung Memorial Hospital and Chang Gung University College of Medicine, Kaohsiung 833, Taiwan; infenit3868@gmail.com; 2 Department of Trauma Surgery, Kaohsiung Chang Gung Memorial Hospital and Chang Gung University College of Medicine, Kaohsiung 833, Taiwan; junyinhaung@yahoo.com.tw (C.-Y.H.); htl1688@yahoo.com.tw (H.-T.L.); 3 Department of Plastic Surgery, Kaohsiung Chang Gung Memorial Hospital and Chang Gung University College of Medicine, Kaohsiung 833, Taiwan; libe320@yahoo.com.tw

**Keywords:** stress-induced hyperglycemia, diabetic hyperglycemia, diabetes mellitus, mortality, intensive care unit

## Abstract

Background: This study was designed to measure the effect of stress-induced hyperglycemia (SIH) and diabetic hyperglycemia (DH) versus non-diabetic normoglycemia (NDN) on the outcomes of trauma patients in the intensive care unit (ICU). Methods: Diabetes mellitus (DM) was determined based on patient history and/or a hemoglobin A1c (HbA1c) level of ≥6.5% at admission. The patients who had serum glucose levels of ≥200 mg/dL in the absence or presence of DM were assigned into the groups SIH and DH, respectively. Diabetic normoglycemia (DN) and NDN were determined based on serum glucose levels of <200 mg/dL in patients with and without DM, respectively. Patients with burn injury or incomplete data were excluded. Detailed data of trauma patients in the ICU of a Level-I trauma center from 1 January 2009 to 31 December 2016 were retrieved from the database of the Trauma Registry System. These patients were classified into four exclusive groups, including NDN (*n* = 1745), DN (*n* = 306), SIH (*n* = 225) and DH (*n* = 206). The Pearson chi-square test was used to compare categorical data between groups. Continuous variables were compared using one-way analysis of variance along with the Games–Howell post hoc test. To decrease the confounding effect of the differences in sex and age, preexisting comorbidities and injury severity score (ISS) among different groups of patients, 1:1 ratio propensity score-matched cohorts were assigned using the NCSS software. The effect of hyperglycemia on the outcomes of patients with and without DM was assessed with a logistic regression analysis. Results: Among those selected propensity score-matched patient cohorts, the patients with SIH and DH had a 3.88-fold (95% CI, 2.13–7.06; *p* < 0.001) and 1.83-fold (95% CI, 1.00–3.34; *p* = 0.048) higher mortality, respectively, than those with NDN. Moreover, the patients in the SIH group (10.0 vs. 7.4 days; *p* = 0.005) and those in the DH group (10.1 vs. 7.4 days; *p* = 0.006) who were admitted to the ICU had a significantly longer length of stay than those in the NDN group. In addition, the SIH group had a 2.13-fold (95% CI, 1.04–4.36; *p* = 0.038) higher adjusted odds ratio for mortality than the DH group. Conclusions: This study revealed significantly worse outcomes in terms of mortality among patients with SIH and DH who were admitted to the ICU after controlling for sex and age, preexisting comorbidities and ISS. In addition, patients who had SIH presented significantly higher adjusted odds for mortality than those DH patients. These results suggest that hyperglycemia is detrimental in patients with or without DM who were admitted to the ICU, and there is a different pathophysiological mechanisms behind the SIH and DH.

## 1. Background

Hyperglycemia is commonly presented among patients in the intensive care unit (ICU). Several published studies have indicated an association between hyperglycemia (a level of serum glucose ≥200 mg/dL) and worse outcomes in trauma patients who were admitted to the ICU [1,2,3,4]. However, not all patients with hyperglycemia had diabetes mellitus (DM), and hyperglycemia may be due to undiagnosed DM or may be secondary to stress [5]. In 2014, there were 21 million individuals presenting with DM and 8.1 million individuals had undiagnosed DM, accounting for 6.7% and 2.6% of the total population, respectively, in the United States [6]. Nearly a quarter of the trauma patients with hyperglycemia were attributed to an undiagnosed DM, which was determined based on an elevated HbA1c level [7]. In a study on 5117 trauma patients, DM was found in 446 patients (8.7%), of which 137 (2.7%) were either diagnosed with occult DM, had an elevated HbA1c level or did not have a history of DM [8]. Of the 137 patients with undiagnosed DM, 85 presented with hyperglycemia, accounting for 16.8% of the patients with hyperglycemia [9].

With the correlation between cortisol and catecholamine levels and injury severity [10], stress-induced hyperglycemia (SIH) commonly occurs in trauma patients who have critical illnesses [9,11,12,13,14,15,16]. The neuroendocrine response to stress can increase the greater adrenal cortical output by 10 times, including excessive glycogenolysis, gluconeogenesis and insulin resistance [17]. An increased level of the pituitary hormone and sympathetic nervous system activation can result in a significant increase in blood glucose levels [18]. Hyperglycemia is believed to be attributed to the insufficient secretion of insulin to cope with the hyperglycemic effect from the catecholamine [19]. Patients with preexisting DM are more susceptible to hyperglycemia due to insulin resistance and subsequent hyperglucagonemia as a natural progression of the disease than in patients without DM during the acute illness [20].

The critically ill nondiabetic hyperglycemic population comprises patients with undiagnosed DM and the patients with SIH [5,21]. The severity of SIH is characterized by its higher rates of morbidity and mortality when compared with those without preexisting DM [13,15,16,22,23]. In the evaluation of the differential effect of SIH versus diabetic hyperglycemia (DH) on the outcomes of trauma patients, a significantly higher mortality rate had been reported in those patients with SIH, but not with DH [8,24,25,26]. The mortality risk was two-fold higher in patients with SIH than the DH patients, whose mortality risk did not significantly increase [8]. Among the selected propensity score-matched patients in terms of all trauma cases, similar results were observed as those patients with SIH had 3.0-fold higher odds ratio of mortality than the patients with non-diabetic normoglycemia (NDN). Nonetheless, patients with DH did not have a significantly increased rate of mortality than those NDN [24]. Few studies have evaluated the differential effect of SIH versus DH on the outcomes of critically ill trauma patients who required ICU admission [8,27]. This study was designed to measure the effect of SIH and DH versus NDN on the outcomes of trauma patients in the ICU under the reduction of the confounding effect of the differences in sex and age, preexisting comorbidities and injury severity among the patient cohorts. Moreover, selected propensity score-matched patients were evaluated for outcome assessment. We hypothesized that patients with SIH who were admitted to the ICU had a worse outcome than those patients with DH.

## 2. Methods

### 2.1. Ethics Statement

The institutional review board (IRB) of the Kaohsiung Chang Gung Memorial Hospital, a Level I regional trauma center in southern Taiwan [28,29], approved this study with Reference Number 201701331B0. The need for informed consent was waived because this is a retrospective study that used data from the registered data of the Trauma Registry System.

### 2.2. Study Population

**Figure 1:**
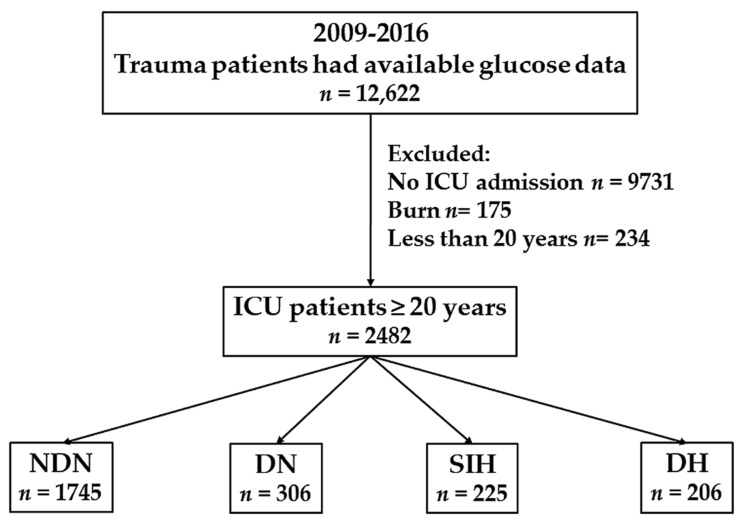
Flowchart of the allocation of patients into the non-diabetic normoglycemia (NDN), diabetic normoglycemia (DN), stress-induced hyperglycemia (SIH) and diabetic hyperglycemia (DH) groups according to the existence of hyperglycemia (serum glucose level ≥200 mg/dL) and DM (patient history and/or admission HbA1c level ≥6.5%).

This study included all adult patients who sustained a trauma injury and were admitted to the ICU from 1 January 2009 to 31 December 2016. This study only included adult patients aged ≥20 years with available data of serum glucose level at the emergency department (ED), as well as a history of DM or measured HbA1c level. Patients with burn injury or incomplete data were excluded. Based on the current recommendations of the American Diabetes Association [30], hyperglycemia is diagnosed when there was a serum glucose level of ≥200 mg/dL at ED, and DM was defined according to patient history and/or hemoglobin A1c (HbA1c) level of ≥6.5% upon or during the first month of admission. Therefore, patients without hyperglycemia, but with DM were assigned to the NDN group, and those without hyperglycemia, but with DM were assigned to the DN group. Moreover, patients who had hyperglycemia with and without DM were assigned to the DH and SIH groups, respectively. The study population was classified into four exclusive groups according to the abovementioned definitions (Figure 1). The retrieved information of the patients included: age; sex; comorbidities, such as coronary artery disease (CAD), congestive heart failure (CHF) and cerebral vascular accident (CVA), hypertension (HTN) and end-stage renal disease (ESRD); serum glucose level upon admission to ED; HbA1c level upon admission to ED or within 1 month of hospitalization; ISS; comorbidities such as pneumonia or acute renal failure (ARF) diagnosed during the course of hospitalization; length of stay (LOS) in the ICU; and mortality in the hospital.

### 2.3. Statistical Analysis

Statistical analysis was performed using Windows Version 22.0 SPSS software (IBM Corp., Armonk, NY, USA). Mortality of the patients in the hospital was the primary outcome of the study. The secondary outcomes included LOS in the ICU and the prevalence rate of pneumonia and ARF during hospitalization. The odds ratios (ORs) with 95% CIs of the associated conditions of the patients were presented. The Levene’s test was used to estimate the homogeneity of variance of the continuous variables. For the continuous variables, the one-way analysis of variance (ANOVA) with the Games–Howell post hoc test was used to evaluate the differences among groups of patients. Continuous data were expressed as the mean ± the standard deviation. The ISS was expressed as the median and interquartile range (IQR, Q1–Q3). To reduce the confounding effects of a non-randomized assignment in assessing outcomes of patients, the NCSS software (NCSS 10; NCSS Statistical Software, Kaysville, UT, USA) was used to create a 1:1 matched patient cohort according to the propensity scores calculated with the greedy method with the following covariates: sex and age, preexisting comorbidities and ISS. The mortality outcomes, as well as the prevalence rates of pneumonia and ARF were assessed using a binary logistic regression model. The LOS in the ICU was calculated with ANOVA. Statistically significance was indicated when there was a *p*-value < 0.05.

## 3. Results

### 3.1. Patient and Injury Characteristics

During the study period, a total of 2482 patients were enrolled in this study (Figure 1) and assigned into four groups: NDN (*n* = 1745), DN (*n* = 306), SIH (*n* = 225) and DH (*n* = 206) (Table 1). Compared with the NDN group, a significant predominant female population was observed in the patients with DN and DH (Table 2). However, in terms of sex, there was no significant difference between the SIH and NDN group. The patients with DN and DH were significantly older than those with NDN. However, no significant difference was found between the patients in the SIH and NDN groups in terms of age. In addition, the patients with SIH were significantly younger than those with DH. The prevalence rates of DN and DH among individuals with comorbidities were significantly higher than those with NDN. However, no significant difference was observed in the SIH group compared with the NDN group in terms of the prevalence rates of comorbidities. The prevalence rates of preexisting comorbidities, such as HTN, CAD and CVA, were significantly lower among patients with SIH than those patients with DH. With a higher ISS, the individuals with SIH (median (IQR, Q1–Q3), 24 [16,17,18,19,20,21,22,23,24,25,26,27,28,29]) had a significantly severe injury compared to those with NDN (16 [13,14,15,16,17,18,19,20,21,22,23,24]), DN (16 [9,10,11,12,13,14,15,16,17,18,19,20]) and DH (17 [14,15,16,17,18,19,20,21,22,23,24,25]). Compared with NDN, DH had a significantly higher ISS. However, DN had a significantly lower ISS. In addition, more patients with SIH had an ISS of ≥25 than those patients in the NDN, DN or DH group.

### 3.2. Outcomes of the Patients

The SIH group had 5.12-fold higher rates of mortality (95% CI, 3.72–7.05; *p* < 0.001) and a significantly longer stay in the ICU (10.1 vs. 6.7 days, respectively; *p* < 0.001) than the NDN group. However, no significant difference was found in the odds for pneumonia and ARF. Compared to NDN, DH had 2.45-fold higher odds for mortality (95% CI, 1.67–3.57; *p* < 0.001), a longer LOS in the ICU (10.1 vs. 6.7 days, respectively; *p* < 0.001) and higher odds for patients to sustain pneumonia complication (OR, 2.43; 95% CI, 1.45–4.07; *p* = 0.001). However, there was no significant difference in terms of the prevalence rate of ARF between the DH and the NDN groups. The DN group did not have a significantly higher odds of mortality than the NDN group; however, the DN group had a longer LOS in the ICU and higher rates of pneumonia (OR, 2.27; 95% CI, 1.45–3.58; *p* < 0.001), as well as ARF (OR, 2.44; 95% CI, 1.06–5.62; *p* = 0.049). Compared to the DH group, the SIH group had significantly higher odds for mortality, but no significant differences were observed in the LOS in the ICU, as well as in the odds for patients with pneumonia and ARF between these two groups of patients.

### 3.3. Outcomes of the Selected Propensity Score-Matched Patients

With the decrease in the effect of the differences in sex and age, preexisting comorbidities and ISS of the patient population on the outcome assessment, patients in propensity score-matched cohorts were selected for further comparison. Using the NDN group as the control, 290, 214 and 200 well-balanced pairs of individuals with DN, SIH and DH, respectively, were selected. Using the DH group as the control, 106 balanced pairs of individuals with SIH were selected. There were no significant differences among the selected pairs of propensity score-matched patients in terms of sex, age, comorbidity and ISS (Table 3). As shown in Table 4, outcomes of the propensity score-matched patients revealed that, compared to the NDN group, the patients with SIH had 3.88-fold higher odds for mortality (95% CI, 2.13–7.06; *p* < 0.001), and the LOS in the ICU was significantly longer (10.0 vs. 7.4 days, respectively; *p* = 0.005). However, no significant differences were observed in the prevalence rates of pneumonia and ARF between the SIH and NDN groups. The patients with DH had a 1.83-fold higher odds for mortality (95% CI, 1.00–3.34; *p* = 0.048), and the LOS in the ICU was significantly longer (10.1 vs. 7.4 days, respectively; *p* = 0.006) than the patients with NDN. However, there were no differences regarding the prevalence rates of pneumonia and ARF between the SIH and NDN groups. Between the NDN and DN groups, the mortality rate, LOS in the ICU or prevalence rates of pneumonia and ARF did not significantly differ. Compared to those patients with DH, the patients with SIH still present 2.13-fold higher odds for mortality (95% CI, 1.04–4.36; *p* = 0.038). No differences were observed in the LOS in the ICU and prevalence rates of pneumonia and ARF between the SIH and DH groups.

## 4. Discussion

Among the patients admitted to the ICU, those with hyperglycemia had a significantly higher mortality rate than those patients with NDN, regardless of whether it was attributed to stress or DM. Even after adjusting the differences in sex and age, comorbidities and injury severity among trauma patients, the mortality rate was 3.88- and 1.83-fold higher in the patients with SIH and DH, respectively, than that in patients with NDN. This result was not in accordance with that observed in hospitalized trauma patients in a study by Kerby et al. [8] and our previous report [26], which demonstrated a significantly higher adjusted mortality for the patients who had SIH, but not for those patients with DH. For the patients with DH, the difference in the outcome of patients staying in the ICU and staying in ward indicates that patients with DM who were critically ill had a worse outcome than those with less severe injury. This study also confirmed the results of previous studies on the different effects of hyperglycemia on patients with and without DM [26,30].

The difference in the observations may be attributed to the use of mortality as the primary outcome. DM-specific microvascular disease leads to blindness, atherosclerosis, nerve damage and renal failure, with an increased risk of myocardial infarction, stroke and limb amputation [31]. Many studies have also revealed the detrimental effects of hyperglycemia on the immune function [32] and infection [12,33]. However, the detrimental effect of hyperglycemia may not significantly affect the prevalence rates of pneumonia and ARF, as well as result in a prolonged stay at the ICU, as shown in this study. Second, the different observations may be due to the different etiologies between trauma patients who need to or need not stay in the ICU. Moreover, hyperglycemia had a differential impact on critically ill patients from different etiological groups [34] because a blood glucose level of >200 mg/dL was associated with mortality in patients admitted to the ICU due to acute myocardial infarction, but not in those with sepsis [34]. In the ICU, more patients were prone to severe traumatic brain injury, as well as chest and abdomen penetration injury. Moreover, these patients were more likely to experience hypovolemic shock than those admitted in the ICU.

Hyperglycemia presents a more deleterious effect on critically ill participants without DM than those with DM [35]. A question frequently asked is whether the preexisting DM may play a protective role in critically ill patients [35]. Patients with DM may be tolerant to degrees of moderate hyperglycemia and therefore may be able to adapt to a high-range fluctuations of glucose levels, whereas patients without DM are less tolerant to even moderate hyperglycemia, thus sustaining an impaired immune defense and perturbation of the microvascular environment, which may lead to organ failure in some cases [5]. Moreover, patients with preexisting DM undergo cellular adaptation to hyperglycemia owing to the reduction of produced reactive oxygen species [36], which is the main molecular mechanism for glucose-mediated vascular damage [37]. In this study, although a higher mortality rate was observed among the patients with SIH or DH than those with NDN who were admitted to the ICU, patients with SIH still had 2.13-fold higher odds of adjusted mortality than those patients with DH. This result suggests that the mechanisms behind the detrimental effects in these two hyperglycemia states in critically ill trauma patients were different. Notably, this study revealed that the mortality rate and LOS in the ICU among patients with DN did not significantly differ compared to those with NDN. Therefore, we did not agree with the assumption regarding the protective role of DM in critically ill patients. However, we prefer the concept that the adverse effect of hyperglycemia is less pronounced in critically ill patients with DM than those without DM.

There were some limitations in this study. First, a retrospective design study may carry a selection bias. Second, the patients declared to be dead at the scene of the accident or upon arrival at the ED were not included in the Trauma Registry System, and this might have resulted in selection bias on mortality outcome assessment. Third, stress might also induce hyperglycemia in patients with DM [38]. Therefore, without the measurement of catecholamine or stress hormone level, the estimation of the hazards ratio of mortality between the SIH and DH groups may be biased. Fourth, the appropriate glucose targets and strategies for sugar control have been inconclusive and may vary among different ICUs. Therefore, we could only assume that all patients had received uniform management in the clinical setting. Finally, the HbA1c level may be not accurate in patients receiving blood transfusions [39], and its value may vary by racial or ethnic group [40]. Thus, it may lead to bias.

## 5. Conclusions

This study revealed that significantly adverse outcomes in terms of mortality were observed among trauma patients with SIH and DH who were admitted to the ICU after controlling for sex and age, preexisting comorbidities and injury severity. In addition, the patients with SIH had significantly higher adjusted odds for mortality than the patients with DH. Clinicians should be aware that patients without DM who were admitted to the ICU are not the only ones who are most likely to have adverse outcomes. In fact, those who were diagnosed with DM may have a significantly worse outcome.

## Figures and Tables

**Table 1:** Epidemiological characteristics of the patients.

Variables	NDN (*n* = 1745)	DN (*n* = 306)	SIH (*n* = 225)	DH (*n* = 206)
Sex				
Male	1144 (65.6)	164 (53.6)	145 (64.4)	117 (56.8)
Female	601 (34.4)	142 (46.4)	80 (35.6)	89 (43.2)
Age	53.3 ± 20.5	69.1 ± 11.8	54.0 ± 17.9	65.5 ± 12.9
Comorbidity				
HTN	407 (23.3)	209 (68.3)	51 (22.7)	128 (62.1)
CAD	79 (4.5)	37 (2.1)	9 (4.0)	29 (14.1)
CHF	8 (0.5)	7 (2.3)	3 (1.3)	6 (2.9)
CVA	60 (3.4)	43 (14.1)	3 (1.3)	20 (9.7)
ESRD	3 (0.2)	0 (0.0)	0 (0.0)	0 (0.0)
ISS, median = (IQR)	16 (13–24)	16 (9–20)	24 (16–29)	17 (14–25)
<16	542 (31.1)	108 (35.3)	40 (17.8)	55 (26.7)
16–24	809 (46.4)	142 (46.4)	75 (33.3)	92 (44.7)
≥25	394 (22.6)	56 (18.3)	110 (48.9)	59 (28.6)
Mortality, *n* (%)	161 (9.2)	27 (8.8)	77 (34.2)	41 (19.9)
ICU LOS (days)	6.7 ± 7.8	8.9 ± 10.9	10.1 ± 12.0	10.1 ± 11.2
Pneumonia	74 (4.2)	28 (9.2)	12 (5.3)	20 (9.7)
ARF	19 (1.1)	8 (2.6)	4 (1.8)	3 (1.5)

HTN = hypertension; CAD = coronary artery disease; CHF = congestive heart failure; CVA = cerebral vascular accident; ESRD = end-stage renal disease; ISS = injury severity score; IQR = interquartile range; ICU = intensive care unit; LOS = length of stay; ARF = acute renal failure; NDN = nondiabetic normoglycemia; DN = diabetic normoglycemia; SIH = stress-induced hyperglycemia; DH = diabetic hyperglycemia.

**Table 2:** Comparison of the characteristics, injury severities and outcomes among the patient groups.

	DN vs. NDN		SIH vs. NDN		DH vs. NDN		SIH vs. DH	
Variables	OR (95% CI)	*p*	OR (95% CI)	*p*	OR (95% CI)	*p*	OR (95% CI)	*p*
Sex		<0.001		0.741		0.013		0.104
Male	0.61 (0.48–0.78)		0.95 (0.71–1.27)		0.69 (0.52–0.93)		1.38 (0.94–2.03)	
Female	1.65 (1.29–2.11)		1.05 (0.79–1.40)		1.45 (1.08–1.94)		0.73 (0.49–1.07)	
Age	-	<0.001	-	0.544	-	<0.001	-	<0.001
Comorbidity								
HTN	7.08 (5.43–9.23)	<0.001	0.96 (0.69–1.34)	0.826	5.40 (3.99–7.30)	<0.001	0.18 (0.12–0.27)	<0.001
CAD	2.90 (1.92–4.38)	<0.001	0.88 (0.44–1.78)	0.719	3.46 (2.20–5.43)	<0.001	0.25 (0.12–0.55)	<0.001
CHF	5.08 (1.83–14.12)	0.003	2.93 (0.77–11.14)	0.122	6.51 (2.24–18.96)	0.002	0.45 (0.11–1.83)	0.321
CVA	4.59 (3.04–6.94)	<0.001	0.38 (0.12–1.22)	0.091	3.02 (1.78–5.12)	<0.001	0.13 (0.04–0.43)	<0.001
ESRD	-	1.000	-	1.000	-	1.000	-	-
ISS, median (IQR)	-	<0.001	-	<0.001	-	0.194	-	<0.001
Mortality, *n* (%)	0.95 (0.62–1.46)	0.822	5.12 (3.72–7.05)	<0.001	2.45 (1.67–3.57)	<0.001	2.09 (1.35–3.25)	0.001
ICU LOS (days)	-	0.001	-	<0.001	-	<0.001	-	0.986
Pneumonia	2.27 (1.45–3.58)	<0.001	1.27 (0.68–2.38)	0.450	2.43 (1.45–4.07)	0.001	0.52 (0.25–1.10)	0.084
ARF	2.44 (1.06–5.62)	0.049	1.64 (0.55–4.88)	0.324	1.34 (0.39–4.58)	0.500	1.23 (0.27–5.54)	1.000

HTN = hypertension; CAD = coronary artery disease; CHF = congestive heart failure; CVA = cerebral vascular accident; ESRD = end-stage renal disease; ISS = injury severity score; IQR = interquartile range; ICU = intensive care unit; LOS = length of stay; ARF = acute renal failure; NDN = nondiabetic normoglycemia; DN = diabetic normoglycemia; SIH = stress-induced hyperglycemia; DH = diabetic hyperglycemia; CI = confidence interval; OR = odds ratio.

**Table 3:** Assessment of covariates in patients adjusted in 1:1 greedy propensity-score matching.

	Propensity-Score Matched Cohort			
DN vs. NDN	DN (*n* = 290)	NDN (*n* = 290)	OR (95% CI)	*p*
Sex			1.00 (0.72–1.39)	1.000
Male	158 (54.5)	158 (54.5)		
Female	132 (45.5)	132 (45.5)		
Age	69.1 ± 11.8	69.1 ± 12.0		0.992
Comorbidity				
HTN	195 (67.2)	195 (67.2)	1.00 (0.71–1.42)	1.000
CAD	27 (9.3)	27 (9.3)	1.00 (0.57–1.75)	1.000
CHF	1 (0.3)	1 (0.3)	1.00 (0.06–16.06)	1.000
CVA	33 (11.4)	33 (11.4)	1.00 (0.60–1.67)	1.000
ESRD	0	0	-	-
ISS, median (IQR)	16 (9–20)	16 (9–20)	-	0.696
**SIH vs. NDN**	**SIH (*n* = 214)**	**NDN (*n* = 214)**	**OR (95% CI)**	***p***
Sex			1.00 (0.67–1.49)	1.000
Male	138 (64.5)	138 (64.5)		
Female	76 (35.5)	76 (35.5)		
Age	53.8 ± 17.6	53.5 ± 17.7	-	0.883
Comorbidity				
HTN	48 (22.4)	48 (22.4)	1.00 (0.64–1.58)	1.000
CAD	6 (2.8)	6 (2.8)	1.00 (0.32–3.15)	1.000
CHF	0 (0.0)	0 (0.0)	-	-
CVA	3 (1.4)	3 (1.4)	1.00 (0.20–5.01)	1.000
ESRD	0 (0.0)	0 (0.0)	-	-
ISS, median (IQR)	24 (16–29)	24 (16–29)	-	0.964
**DH vs. NDN**	**DH (*n* = 200)**	**NDN (*n* = 200)**	**OR (95% CI)**	***p***
Sex			1.00 (0.67–1.49)	1.000
Male	115 (57.5)	115 (57.5)		
Female	85 (42.5)	85 (42.5)		
Age	65.4 ± 13.0	65.8 ± 13.3	-	0.767
Comorbidity				
HTN	124 (62.0)	124 (62.0)	1.00 (0.67–1.50)	1.000
CAD	27 (13.5)	27 (13.5)	1.00 (0.56–1.78)	1.000
CHF	1 (0.5)	1 (0.5)	1.00 (0.06–16.10)	1.000
CVA	20 (10.0)	20 (10.0)	1.00 (0.52–1.92)	1.000
ESRD	0 (0.0)	0 (0.0)	-	-
ISS, median (IQR)	17 (14–25)	17 (13.25–25)	-	0.767
**SIH vs. DH**	**SIH (*n* = 106)**	**DH (*n* = 106)**	**OR (95% CI)**	***p***
Sex			1.00 (0.58–1.72)	1.000
Male	61 (57.5)	61 (57.5)		
Female	45 (42.5)	45 (42.5)		
Age	61.8 ± 15.0	61.5 ± 13.9	-	0.872
Comorbidity				
HTN	42 (39.6)	42 (39.6)	1.00 (0.58–1.73)	1.000
CAD	5 (4.7)	5 (4.7)	1.00 (0.28–3.56)	1.000
CHF	0 (0.0)	0 (0.0)	-	-
CVA	3 (2.8)	3 (2.8)	1.00 (0.20–5.07)	1.000
ESRD	0 (0.0)	0 (0.0)	-	-
ISS, median (IQR)	20 (16–25)	20 (16–25)	-	0.998

HTN = hypertension; CAD = coronary artery disease; CHF = congestive heart failure; CVA = cerebral vascular accident; ESRD = end-stage renal disease; ISS = injury severity score; IQR = interquartile range; ICU = intensive care unit; LOS = length of stay; NDN = nondiabetic normoglycemia; DN = diabetic normoglycemia; SIH = stress-induced hyperglycemia; DH = diabetic hyperglycemia.

**Table 4:** Outcomes comparison among the selected propensity score-matched patients.

	Propensity-Score Matched Cohort			
**DN vs. NDN**	**DN (*n* = 290)**	**NDN (*n* = 290)**	**OR (95% CI)**	***p***
Mortality, *n* (%)	26 (9.0)	33 (11.4)	0.70 (0.39–1.25)	0.226
ICU LOS (days)	8.5 ± 10.2	7.3 ± 8.0	-	0.108
Pneumonia	26 (9.0)	18 (6.2)	1.47 (0.78–2.75)	0.232
ARF	7 (2.4)	2 (0.7)	3.77 (0.76–18.63)	0.103
**SIH vs. NDN**	**SIH (*n* = 214)**	**NDN (*n* = 214)**	**OR (95% CI)**	***p***
Mortality, *n* (%)	69 (32.2)	30 (14.0)	3.88 (2.13–7.06)	<0.001
ICU LOS (days)	10.0 ± 11.6	7.4 ± 6.8	-	0.005
Pneumonia	12 (5.6)	12 (5.6)	1.24 (0.48–3.19)	0.651
ARF	4 (1.9)	3 (1.4)	0.75 (0.15–3.70)	0.723
**DH vs. NDN**	**DH (*n* = 200)**	**NDN (*n* = 200)**	**OR (95% CI)**	***p***
Mortality, *n* (%)	39 (19.5)	25 (12.5)	1.83 (1.00–3.34)	0.048
ICU LOS (days)	10.1 ± 11.2	7.4 ± 8.3	-	0.006
Pneumonia	19 (9.5)	13 (6.5)	1.57 (0.70–3.52)	0.270
ARF	2 (1.0)	2 (1.0)	0.66 (0.09–5.08)	0.691
**SIH vs. DH**	**SIH (*n* = 106)**	**DH (*n* = 106)**	**OR (95% CI)**	***p***
Mortality, *n* (%)	33 (31.1)	20 (18.9)	2.13 (1.04–4.36)	0.038
ICU LOS (days)	10.3 ± 12.9	9.0 ± 9.2	-	0.395
Pneumonia	6 (5.7)	10 (9.4)	0.64 (0.21–1.94)	0.425
ARF	1 (0.9)	2 (1.9)	0.58 (0.05–6.59)	0.658

HTN = hypertension; CAD = coronary artery disease; CHF = congestive heart failure; CVA = cerebral vascular accident; ESRD = end-stage renal disease; ISS = injury severity score; IQR = interquartile range; ICU = intensive care unit; LOS = length of stay; ARF = acute renal failure; NDN = nondiabetic normoglycemia; DN = diabetic normoglycemia; SIH = stress-induced hyperglycemia; DH = diabetic hyperglycemia; CI = confidence interval; OR = odds ratio.

## References

[1] Vanhorebeek I., Langouche L., Van den Berghe G. (2007). Tight blood glucose control with insulin in the ICU: Facts and controversies. Chest.

[2] Sechterberger M.K., van Steen S.C., Boerboom E.M., van der Voort P.H., Bosman R.J., Hoekstra J.B., DeVries J.H. (2017). Higher glucose variability in type 1 than in type 2 diabetes patients admitted to the intensive care unit: A retrospective cohort study. J. Crit. Care.

[3] Ali Abdelhamid Y., Kar P., Finnis M.E., Phillips L.K., Plummer M.P., Shaw J.E., Horowitz M., Deane A.M. (2016). Stress hyperglycaemia in critically ill patients and the subsequent risk of diabetes: A systematic review and meta-analysis. Crit. Care.

[4] Santos L. (2013). Stress response in critical illness. Curr. Probl. Pediatr. Adolesc. Health Care.

[5] Smith F.G., Sheehy A.M., Vincent J.L., Coursin D.B. (2010). Critical illness-induced dysglycaemia: Diabetes and beyond. Crit. Care.

[6] Centers for Disease Control and Prevention (2014). National Diabetes Statistics Report: Estimates of Diabetes and Its Burden in the United States.

[7] Kopelman T.R., O’Neill P.J., Kanneganti S.R., Davis K.M., Drachman D.A. (2008). The relationship of plasma glucose and glycosylated hemoglobin A1C levels among nondiabetic trauma patients. J. Trauma.

[8] Kerby J.D., Griffin R.L., MacLennan P., Rue L.W. (2012). Stress-induced hyperglycemia, not diabetic hyperglycemia, is associated with higher mortality in trauma. Ann. Surg..

[9] Richards J.E., Kauffmann R.M., Obremskey W.T., May A.K. (2013). Stress-induced hyperglycemia as a risk factor for surgical-site infection in nondiabetic orthopedic trauma patients admitted to the intensive care unit. J. Orthop. Trauma.

[10] Marik P.E. (2009). Critical illness-related corticosteroid insufficiency. Chest.

[11] Vogelzang M., van der Horst I.C., Nijsten M.W. (2004). Hyperglycaemic index as a tool to assess glucose control: A retrospective study. Crit. Care.

[12] Yendamuri S., Fulda G.J., Tinkoff G.H. (2003). Admission hyperglycemia as a prognostic indicator in trauma. J. Trauma.

[13] Sung J., Bochicchio G.V., Joshi M., Bochicchio K., Tracy K., Scalea T.M. (2005). Admission hyperglycemia is predictive of outcome in critically ill trauma patients. J. Trauma.

[14] Richards J.E., Kauffmann R.M., Zuckerman S.L., Obremskey W.T., May A.K. (2012). Relationship of hyperglycemia and surgical-site infection in orthopaedic surgery. J. Bone Joint Surg. Am..

[15] Mraovic B., Suh D., Jacovides C., Parvizi J. (2011). Perioperative hyperglycemia and postoperative infection after lower limb arthroplasty. J. Diabetes Sci. Technol..

[16] Leto R., Desruelles D., Gillet J.B., Sabbe M.B. (2015). Admission hyperglycaemia is associated with higher mortality in patients with hip fracture. Eur. J. Emerg. Med..

[17] Marik P.E., Bellomo R. (2013). Stress hyperglycemia: An essential survival response!. Crit. Care.

[18] Desborough J.P. (2000). The stress response to trauma and surgery. Br. J. Anaesth..

[19] Harp J.B., Yancopoulos G.D., Gromada J. (2016). Glucagon orchestrates stress-induced hyperglycaemia. Diabetes Obes. Metab..

[20] Silva-Perez L.J., Benitez-Lopez M.A., Varon J., Surani S. (2017). Management of critically ill patients with diabetes. World J. Diabetes.

[21] Clement S., Braithwaite S.S., Magee M.F., Ahmann A., Smith E.P., Schafer R.G., Hirsch I.B. (2004). Management of diabetes and hyperglycemia in hospitals. Diabetes Care.

[22] Bosarge P.L., Kerby J.D. (2013). Stress-induced hyperglycemia: Is it harmful following trauma?. Adv. Surg..

[23] Ray B., Ludwig A., Yearout L.K., Thompson D.M., Bohnstedt B.N. (2017). Stress-Induced Hyperglycemia After Spontaneous Subarachnoid Hemorrhage and Its Role in Predicting Cerebrospinal Fluid Diversion. World Neurosurg..

[24] Rau C.S., Wu S.C., Chen Y.C., Chien P.C., Hsieh H.Y., Kuo P.J., Hsieh C.H. (2017). Higher Mortality in Trauma Patients Is Associated with Stress-Induced Hyperglycemia, but Not Diabetic Hyperglycemia: A Cross-Sectional Analysis Based on a Propensity-Score Matching Approach. Int. J. Environ. Res. Public Health.

[25] Rau C.S., Wu S.C., Chen Y.C., Chien P.C., Hsieh H.Y., Kuo P.J., Hsieh C.H. (2017). Mortality Rate Associated with Admission Hyperglycemia in Traumatic Femoral Fracture Patients Is Greater Than Non-Diabetic Normoglycemic Patients but Not Diabetic Normoglycemic Patients. Int. J. Environ. Res. Public Health.

[26] Rau C.S., Wu S.C., Chen Y.C., Chien P.C., Hsieh H.Y., Kuo P.J., Hsieh C.H. (2017). Stress-Induced Hyperglycemia, but Not Diabetic Hyperglycemia, Is Associated with Higher Mortality in Patients with Isolated Moderate and Severe Traumatic Brain Injury: Analysis of a Propensity Score-Matched Population. Int. J. Environ. Res. Public Health.

[27] Lionel K.R., John J., Sen N. (2014). Glycated hemoglobin A: A predictor of outcome in trauma admissions to intensive care unit. Indian J. Crit. Care Med..

[28] Hsieh C.H., Hsu S.Y., Hsieh H.Y., Chen Y.C. (2017). Differences between the sexes in motorcycle-related injuries and fatalities at a Taiwanese level I trauma center. Biomed. J..

[29] Hsieh C.H., Liu H.T., Hsu S.Y., Hsieh H.Y., Chen Y.C. (2017). Motorcycle-related hospitalizations of the elderly. Biomed. J..

[30] Graham B.B., Keniston A., Gajic O., Trillo Alvarez C.A., Medvedev S., Douglas I.S. (2010). Diabetes mellitus does not adversely affect outcomes from a critical illness. Crit. Care Med..

[31] American Diabetes Association (2012). Diagnosis and classification of diabetes mellitus. Diabetes Care.

[32] Khaodhiar L., McCowen K., Bistrian B. (1999). Perioperative hyperglycemia, infection or risk?. Curr. Opin. Clin. Nutr. Metab. Care.

[33] Karunakar M.A., Staples K.S. (2010). Does stress-induced hyperglycemia increase the risk of perioperative infectious complications in orthopaedic trauma patients?. J. Orthop. Trauma.

[34] Wernly B., Lichtenauer M., Franz M., Kabisch B., Muessig J., Masyuk M., Kelm M., Hoppe U.C., Jung C. (2016). Differential Impact of Hyperglycemia in Critically Ill Patients: Significance in Acute Myocardial Infarction but Not in Sepsis?. Int. J. Mol. Sci..

[35] Krinsley J.S. (2010). Moving closer to untangling a sweet web: Hyperglycemia, diabetic status, and mortality in the critically ill. Crit. Care Med..

[36] Dugan L.L., You Y.H., Ali S.S., Diamond-Stanic M., Miyamoto S., DeCleves A.E., Andreyev A., Quach T., Ly S., Shekhtman G. (2013). AMPK dysregulation promotes diabetes-related reduction of superoxide and mitochondrial function. J. Clin. Investig..

[37] Brownlee M. (2001). Biochemistry and molecular cell biology of diabetic complications. Nature.

[38] Rau C.S., Wu S.C., Chen Y.C., Chien P.C., Hsieh H.Y., Kuo P.J., Hsieh C.H. (2017). Stress-Induced Hyperglycemia in Diabetes: A Cross-Sectional Analysis to Explore the Definition Based on the Trauma Registry Data. Int. J. Environ. Res. Public Health.

[39] The International Expert Committee (2009). International Expert Committee report on the role of the A1C assay in the diagnosis of diabetes. Diabetes Care.

[40] Ziemer D.C., Kolm P., Weintraub W.S., Vaccarino V., Rhee M.K., Twombly J.G., Narayan K.M., Koch D.D., Phillips L.S. (2010). Glucose-independent, black-white differences in hemoglobin A1c levels: A cross-sectional analysis of 2 studies. Ann. Intern. Med..

